# Tetrahydrocurcumin regulates the tumor immune microenvironment to inhibit breast cancer proliferation and metastasis via the CYP1A1/NF-κB signaling pathway

**DOI:** 10.1186/s12935-023-02850-9

**Published:** 2023-01-27

**Authors:** Anqi Zeng, Xinyue Yu, Bao Chen, Lu Hao, Ping Chen, Xue Chen, Yuan Tian, Jing Zeng, Hua Hua, Ying Dai, Junning Zhao

**Affiliations:** 1 Sichuan Academy of Traditional Chinese Medicine, Chengdu, 610041 Sichuan China; 2 Sichuan Institute for Translational Chinese Medicine, Chengdu, 610041 Sichuan China; 3 West China School of Pharmacy, Sichuan University, Chengdu, 610041 Sichuan China; 4 School of Pharmacy, Southwest Medical University, Luzhou, 646000 Sichuan China

**Keywords:** Tetrahydrocurcumin, Tumor immune microenvironment, Breast cancer, Proliferation, Metastasis, CYP1A1, NF-κB

## Abstract

The NF-κB signaling pathway is overactivated in tumor cells, and the activation of the NF-κB signaling pathway releases a large number of inflammatory factors, which enhance tumor immunosuppression and promote tumor metastasis. The cytochrome P450 (CYP450) system consists of important metabolic enzymes present in different tissues and progressive tumors, which may lead to changes in the pharmacological action of drugs in inflammatory diseases such as tumors. In this study, the anticancer effect of tetrahydrocurcumin (THC), an active metabolite of curcumin, on breast cancer cells and the underlying mechanism were investigated. Result showed that THC selectively inhibited proliferation and triggered apoptosis in breast cancer cells in a concentration- and time-dependent manner. Moreover, THC-induced cell apoptosis via a mitochondria-mediated pathway, as indicated by the upregulated ratio of Bax/Bcl-2 and reactive oxygen species (ROS) induction. In addition, THC could affect the CYP450 enzyme metabolic pathway and inhibit the expression of CYP1A1 and activation of the NF-κB pathway, thereby inhibiting the migration and invasion of breast cancer cells. Furthermore, after overexpression of CYP1A1, the inhibitory effects of THC on the proliferation, metastasis, and induction of apoptosis in breast cancer cells were weakened. The knockdown of CYP1A1 significantly enhanced the inhibitory effect of THC on the proliferation, metastasis, and apoptosis induction of breast cancer cells. Notably, THC exhibited a significant tumor growth inhibition and anti-pulmonary metastasis effect in a tumor mouse model of MCF-7 and 4T1 cells by regulating the tumor immunosuppressive microenvironment. Collectively, these results showed that TH could effectively trigger apoptosis and inhibit the migration of breast cancer cells via the CYP1A1/NF-κB signaling pathway, indicating that THC serves as a potential candidate drug for the treatment of breast cancer.

## Introduction

Breast cancer is the common malignancy worldwide and the second leading cause of cancer death in women [1]. Statistics show that the incidence of breast cancer is increasing rapidly worldwide [2–4]. Tetrahydrocurcumin (THC) is an important metabolite of curcumin in vivo, and THC has attracted considerable attention because of its good stability and safety. Studies have shown that THC has more desirable biological and pharmacological properties than curcumin [5]. It is considered to be an advanced antioxidant with chemoprophylaxis, showing positive therapeutic effects on a variety of diseases, including hypertension, atherosclerosis, diabetes, neurotoxicity, cardiovascular disease, hepatotoxicity, and liver fibrosis [6, 7]. THC has also been proven useful in the prevention and treatment of many types of cancer. Studies have confirmed that THC has a better and safer effect than curcumin in liver cancer, breast cancer, and other tumors [8–10].

The cytochrome P450 (CYP450) system consists of important metabolic enzymes present in different tissues. The expression of CYPs is regulated by cytokines during information transmission, which may lead to changes in the pharmacological action of substances in inflammatory diseases, such as cancer. The CYP family is involved in the metabolism of many carcinogens, producing secondary metabolites, which can lead to DNA damage and chemical admixtures. It can also be used for the activation or inactivation of anticancer drugs [11, 12]. CYP1A1, CYP1A2, and CYP2E1 can reveal the association between gene polymorphism and cancer susceptibility, and they are responsible for the biotransformation of chemical substances [13]. Studies have shown that the activation of the NF-κB signaling pathway is involved in the regulation of CYP1A1 by heavy metals. Current studies have found that the NF-κB pathway plays a role in almost all processes of tumor genesis, development, migration, invasion, proliferation, and death [14]. Immunity is associated with inflammation, and controlling the inflammatory process is a main activity of NF-κB protein. In addition, NF-κB plays a key role in the immune response, and it is closely related to the tumor [15, 16]. Tumor microenvironment plays an important role in tumor malignant progression, immune escape, and treatment resistance.

In this study, we investigated the effect of THC on breast cancer cells MCF-7 and 4T1. Consequently, we found that THC exhibited its anticancer activity by regulating the tumor immune microenvironment (TIME) to inhibit breast cancer proliferation and metastasis in a dose-dependent manner. Subsequently, we demonstrated for the first time that THC regulated TIME to inhibit breast cancer proliferation and metastasis through the CYP1A1/NF-κB signaling pathway in vitro and in vivo.

## Materials and methods

### Cell culture

MCF-7, MDA-MB-231, MDA-MB-453 and 4T1 cell lines were obtained from the Cell Bank of the Chinese Academy of Sciences. 4T1-Luciferase cells were obtained from Guangzhou Cellcook Biotechnology Co., Ltd. All cells were maintained in MEM and RPMI-1640 medium with 10% fetal bovine serum, 100 units/mL of penicillin, and 100 mg/mL of streptomycin comprising 5% CO_2_ controlling the temperature at 37 ℃.

### Reagents and antibodies

Antibodies against cyclin D1, cyclin B1, CDK1, p21, Bax, Bcl-2, PARP, p-PARP, Cleaved caspase-3, caspase-3, E-Cadherin, N-Cadherin, Vimentin, NF-κB, IκBα, p-IκBα, PD-L1 and β-actin were purchased from Cell Signaling Technology. Anti-Ki-67 mouse monoclonal antibody was obtained from Merck Millipore.

### Cell viability assay

The cell viability of MCF-7 and 4T1 with THC treatments was determined using cell counting kit-8 (CCK8) (BOSTER, Cat.: AR1160). About 3–5 × 10^3^ cells per well were seeded into 96-well plates and then treated with various concentrations of THC with or without inhibitor or inducer for 24, 48, and 72 h. After treatment, 100 mL per well medium, including 10% CCK-8, was added in wells and incubated at 37 ℃ for 1–4 h and then measured at 450 nm wavelength.

### Colony formation assay

A colony formation assay was measured as previously described. In brief, 4T1 and MCF-7 cells were seeded in specified numbers (300–1000 cells/well) in six-well plates. After 24 h incubation, the cells were treated with various concentrations of THC and then incubated for additional 12 days. Then, the cells were fixed with methanol and stained with a 0.01% crystal violet solution for 30 min, and the colonies (600 cells) were counted under a microscope. Data shown represent the average of three independent experiments.

### EDU experiment

After the cells grew to fusion, they were seeded in six-well plates at a density of 1 × 10^5^ cells/well. After the cells were cultured overnight, they were treated with THC at different concentrations for 48 h. EDU working fluid was prepared according to the instruction. The EDU working solution was added to each well. After removing the culture medium, 4% paraformaldehyde fixative was added to each well and fixed. After removing the permeability fluid, washing solution was used to clean the cells. Click additive solution was prepared according to the instruction. After preparation, the solution was divided and stored in a refrigerator set at − 20 ℃. Liquid was washed in step. Click reaction solution was added to each well, and the culture plate was gently shaken to ensure that the reaction mixture covered the sample evenly. Click reaction solution was removed, and the cells were washed. Fluorescence was detected under a microscope.

### Cell cycle detection

The cells at the logarithmic growth stage were digested with trypsin and blown into single cells in a culture medium, and then they were suspended in a complete culture medium for reserve. Six-well plates were prepared, and the cells were inoculated in plates containing a culture medium at a density of 1 × 10^5^ cells per well. On the next day, the prepared THC solution was added to the corresponding wells and then incubated in an incubator. The cells in each group were digested with trypsin and blown into single cells. The cells were collected into a 15 mL centrifuge tube. The cells were washed with phosphate-buffered saline (PBS). After being fixed, the cells were centrifuged, and the supernatant was discarded. Then the cells were re-suspended by adding the configured PI solution. The cells were incubated and detected by flow cytometry (FCM) [17].

### Western blot analysis

About 6 × 10^5^ cells were added in 100 mm dishes. After attachment, the cells were given different treatments for 48 h. Then the collected cells were lysed in RIPA (Beyotime Biotechnology, P0013B) for 30 min on ice. After centrifugation at 10,000 rpm (Thermo Fisher, ST16R), the supernatant was collected, and protein concentrations were quantified by using the BCA Protein Assay Kit (Beyotime Biotechnology, P0010). Equal amounts of total protein were resolved in 10% SDS-PAGE and then transferred to PVDF membranes (Millipore, ISEQ00010). Membranes with proteins were used for blocking, washing, antibody incubation, and finally detection with enhanced chemiluminescence.

### Measurement of ROS level in cells

About 3 × 10^5^ cells were added in 60 mm plates. After attachment overnight, cells were treated differently for 48 h and then replaced with serum-free medium containing 10 mmol/L of DCFH-DA (Beyotime, S0033) for 0.5 h. Cells were washed two times with PBS to remove remnant DCFH-DA. Then, the cells were re-suspended in 300 mL of PBS, and the ROS generation was analyzed by FCM with about 10,000 labeled cells analyzed [18].

### Apoptosis assays

The percentage of apoptosis in cells was assayed in accordance with the instructions (BD, 556,547). About 2 × 10^5^ cells were added in 60 mm dishes and given different treatments. After treatment for 48 h, the cells were collected and re-suspended in 300 mL of ice-cold 1 × binding buffer and dyed with PI and FITC Annexin V for about 30 min at 4 ℃ in the dark. The results were analyzed by using a flow cytometer.

### ΔΨm assay

Rh123 was used to test the changes in ΔΨm by FCM. In brief, cells (1–2 × 10^5^ cells per well) were seeded in a six-well plate overnight and treated with THC for 24 h, and then the harvested cells were washed with cold PBS and incubated with Rh123 solution (5 μg/mL) at 37 °C for 30 min in the dark. ΔΨm was measured by FCM [18].

### Wound-healing migration assay

Wound-healing migration assay was performed as previously described. When the cancer cells grew to 90% confluence, the cell monolayer was scraped by sterile 0.1 mL pipette tips, and fresh medium containing only 1% FBS was added containing different concentrations of THC. After 48 h incubation, cells were fixed and photographed. Images were acquired using a microscope (Leica, DMi1), and the percentage inhibition of migrated cells was expressed using 100% as the value assigned for the untreated group [17]. Inhibitory rate = (W_0h_–W_c_)/(W_0h_–W_0_) × 100%, where W_0h_ represents wound width in 0 h, W_c_ represents wound width in 48 h in after treated with corresponded concentration of THC, W_0_ represents wound width in 48 h after controlled treatment.

### Boyden chamber migration and invasion assay

For the evaluation of cell migration, 8 mm pore-size culture inserts (Transwell, Costar) were placed into the wells of 24-well culture plates, separating the upper and lower chambers. In the upper chamber, 5 × 10^4^ cells suspended in 300 μL of serum-free medium containing different concentrations of THC were added, and 500 μL of culture medium containing 10% FBS was added to the lower chamber. For Matrigel invasion assays, 1 × 10^5^ cells were added to the upper chamber pre-coated with Matrigel, containing different concentrations of THC. After incubation for 48 h at 37 °C, non-migrated and non-invasive cells located on the upper surface of the filter were removed using a cotton swab, and the migrated and invasive cells on the bottom surface of the membrane were fixed with methanol and stained with 0.1% crystal violet. Finally, the cells were imaged and counted using a microscope (Olympus) [17].

### Transcriptome analysis

After digestion, the cells of each group were re-suspended, and the cells were suspended in the medium for later use. Six-well plates were prepared, and the cells were inoculated into six-well plates containing 2.00 mL of culture medium at a density of 1 × 10^6^ cells per well. In addition, the culture plates were shaken using the method shown in Fig. 8. Then cells were cultured overnight in a 37 ℃ incubator. The next day, THC solution was added and cultured for 48 h. After digestion and centrifugation, the cells were re-suspended. After adding and mixing 1 mL of TRIzol, the cells were sent to Oebiotech Biological Company for transcriptome sequencing [15].

### Plasmid synthesis

Plasmids were synthesized by using the Engine organism.

### Transfection plasmid

Breast cancer cell lines MCF-7 and 4T1 were divided into the blank control group, no-load plasmid group, and transfection group. The transfection group was transfected with overexpressed or knockdown plasmids to increase or decrease the CYP1A1 expression, whereas the empty plasmid group was transfected with empty plasmids. Cells at the logarithmic growth stage were collected and inoculated into six-well plates at a concentration of 1 × 10^5^ cells/well. When the cells were cultured to a 90–95% degree of integration, the medium in six-well plates was replaced with 2 mL of serum-free medium. Ten microliters of Lipofectamine 2000 were added to 240 μL of serum-free medium (total volume of 250 μL) and incubated at room temperature for 5 min. Then 4 μg of corresponding plasmid DNA was added to 246 μL of serum-free medium (total volume of 250 μL) and incubated at room temperature for 5 min. Lipofectamine 2000 solution and plasmid DNA solution were mixed evenly and incubated at room temperature for 20 min. The mixed solution of Lipofectamine 2000 and plasmid DNA was added dropwise into the cell holes containing 2 mL of serum-free medium and incubated in a cell incubator for 6 h. After 6 h of culture, the solution in the well plate was replaced with a cell complete medium, and the culture was placed in the cell incubator for 24 h. Finally, Western blot (WB) assay was used to detect whether the transfection was successful.

### Immunofluorescence assay

MCF-7 cells were cultured on glass circles in 24-well plates and exposed to different treatments. Next, cells were fixed with 4% paraformaldehyde for 10 min, incubated with permeabilization solution (1% Triton X-100) for 15 min, washed with cold PBS three times, and blocked with 5% BSA solution for 1 h. Cells were incubated with primary antibodies overnight at 4 °C, washed with cold PBS, and then incubated with FITC-conjugated goat anti-rabbit IgG secondary antibody (dilution, 1:500, Beyotime) for 1 h at 37 °C. Finally, the nuclei of MCF-7 cells were stained with DAPI (dilution, 1:10,000) for 10 min, and cell images were obtained using a laser scanning confocal microscope (Leica) [16].

### Molecular docking

Small molecular ligands file was downloaded by using the PubChem database (https://pubchem.ncbi.nlm.nih.gov/) and imported it into Chem3D after spatial structure transformation and energy optimization software. The output format file is mol2. After processing by Auto Dock Tools 1.5.6, the file is saved as pdbqt format. The gene ID of the core target was retrieved from the Uniprot database and stored in the PDB database (http://www1.rcsb.org/) download the corresponding PDB format file, and carry out the water molecule removal and ligand separation through Pymol software. The macromolecular receptor files of the obtained core targets were imported into Auto Dock Tools 1.5.6 software for hydroprocessing and saved in pdbqt format. Autodock vina 1.1.2 software was used for molecular docking of the core target and its corresponding chemical components, and binding energy was used as the docking evaluation index.

### In vivo antitumor evaluation

All animal experiments were approved by the Institutional Animal Care and Treatment Committee of Laboratory Animal Center of Sichuan Academy of Chinese Medicine, and were conducted in accordance with ARRIVE guidelines. Female BALB/c nude mice and BALB/c mice (6–8 weeks old) were purchased from HFK Bioscience CO., Ltd. These BALB/c mice or BALB/c nude mice were inoculated with 100 μL of 4T1 cell suspension (1 × 10^6^ cells) or MCF-7 cell suspension (1.0 × 10^7^ cells). On the fifth day after inoculation, when the tumor volume reached approximately 100 mm^3^, the mice were equally and randomly divided into three treatment groups (vehicle, 80, and 160 mg/kg; n = 6). Mice received THC treatment by intraperitoneal injection once daily for 2 weeks. The tumor volume (0.5 × L × W^2^, where L is length and W is width) and body weight of the mice were recorded every 2 days. The mice were euthanized by cervical dislocation at the end of the experiments. The tumors were isolated, imaged, weighed, and fixed with paraformaldehyde for further immunohistochemistry evaluation.

### Anti-metastasis evaluation

The anti-metastatic effect of THC was evaluated in a lung metastatic 4T1 model. 1 × 10^6^ 4T1-luciferase cells were injected into BALB/c mice via the tail vein to establish the pulmonary metastasis model. A total of 18 mice were equally and randomly divided into three groups (vehicle, 80, and 160 mg/kg). The mice received THC treatment 2 days after inoculation via intraperitoneal injection once daily for 2 weeks. At the determined time (5, 10, or 15 days after treatment), the mice were anesthetized and intraperitoneally administered with 100 μL of d-luciferin (30 mg/mL in PBS), and bioluminescence from metastatic lung tumors was analyzed using an IVIS Lumina in vivo Imaging System (PerkinElmer). At the end of the experiment, the lungs were isolated from the euthanized mice, and the number of metastatic nodules was counted; nodules were fixed with paraformaldehyde for further evaluation using hematoxylin and eosin (H&E) staining.

### TIME

Single-cell suspensions of tumors from different treated groups were prepared and stained with various antibodies to investigate the effect of THC on the tumor microenvironment (CD3, CD4, CD8, CD11b, Gr-1, F4/80, and CD206) and analyze the population of CD4 + T cells, CD8 + T cells, and myeloid-derived suppressor cells (MDSC) by FCM.

### Statistical analysis

Unless otherwise stated, all experiments were performed for at least three times. The results were expressed in mean ± standard deviation. The statistical significance of the results was determined by *t*-test in GraphPad Prism.

## Results

### THCs inhibited the proliferation of breast cancer cells in vitro

**Fig. 1:**
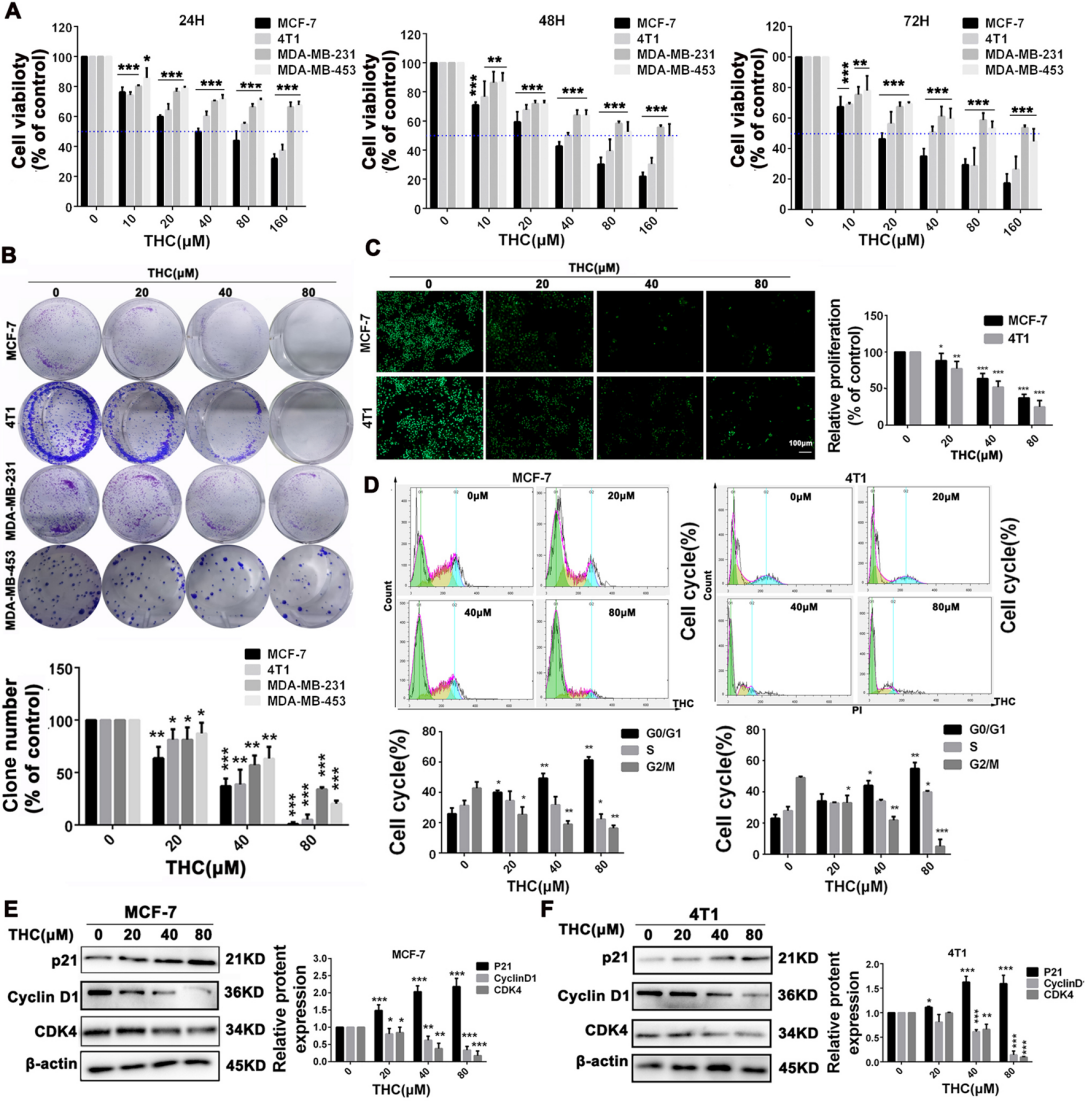
Effects of THC on proliferation and cell cycle of breast cancer cells. **A** Proliferation of MCF-7, MDA-MB-231, MDA-MB-453 and 4T1 cells treated with various concentrations (0, 10, 20, 40, 80, 160 μM) of THC for 24, 48 and 72 h, respectively. Cell viability was detected by CCK8 assay. **B** The effects of THC (0, 20, 40, 80 μM) on colony formation in MCF-7, MDA-MB-231, MDA-MB-453 and 4T1 cells for about 12 days, the statistic results of colony formation assays presented as surviving colonies. Data are expressed as means SD from three experiments. **C** EDU results of MCF-7 and 4T1 cells after treatment with different dose (0, 20, 40, 80 μM) of THC. **D** H1299 cells were exposed to various concentrations of THC (0, 20, 40, 80 μM) for 48 h followed by analysis of cell cycle by flow cytometry and distribution of MCF-7, 4T1cells at different phases of the cell cycle distribution. **E**–**F** The change of p21, CDK4, and CyclinD1 expression. MCF-7 and 4T1cells were treated with different concentrations of THC. After 48 h, cells were harvested, and western blot assay was conducted to test the expression of p21, CDK4, and CyclinD1. β-actin served as loading control. Data were expressed as mean ± SD. *P < 0.05, **P < 0.01, ***P < 0.001 compared to control

Four breast cancer cell lines, namely, MCF-7, MDA-MB-231, MDA-MB-453, and 4T1, were treated with different concentrations of THC for 24, 48, and 72 h to investigate the effect of THC. Consequently, the CCK-8 assay showed THC-induced cell death in breast cancer in a dose-dependent manner (Fig. 1A). The effects of THC on the clonogenesis of breast cancer cells were further studied by plate clonogenesis assay. After 12 days of culture, the results showed (Fig. 1B) that THC could significantly inhibit the formation of MCF-7 and 4T1 clonal colonies of breast cancer cells in a concentration-dependent manner. The proliferating cells could be accurately determined by EDU staining. The concentrations of THC were 0, 20, 40, and 80 μM. The experimental results show (Fig. 1C) that compared with the blank control group, the proliferation activity of MCF-7 cells was significantly inhibited after high-dose THC treatment. Similar results were found in 4T1 cells. These results indicate that high-dose THC can inhibit the proliferation of breast cancer cells. In addition, FCM showed (Fig. 1D) that THC treatment of breast cancer cells resulted in the accumulation of G0/G1 phase cells in MCF-7 and 4T1 cells, and the number of cells increased in this cycle in a concentration-dependent manner. The number of cells in S and G2/M phases decreased, which reduced the number of cells undergoing division. Cell cycle–related proteins were detected by WB assay, and the experimental results showed (Fig. 1E) that THC increased the expression level of p21 protein in MCF-7 and 4T1 cells and decreased the expression level of CyclinD1 and CDK4 proteins.

### THCs promoted the apoptosis of breast cancer cells through the mitochondrial apoptotic pathway in vitro

**Fig. 2:**
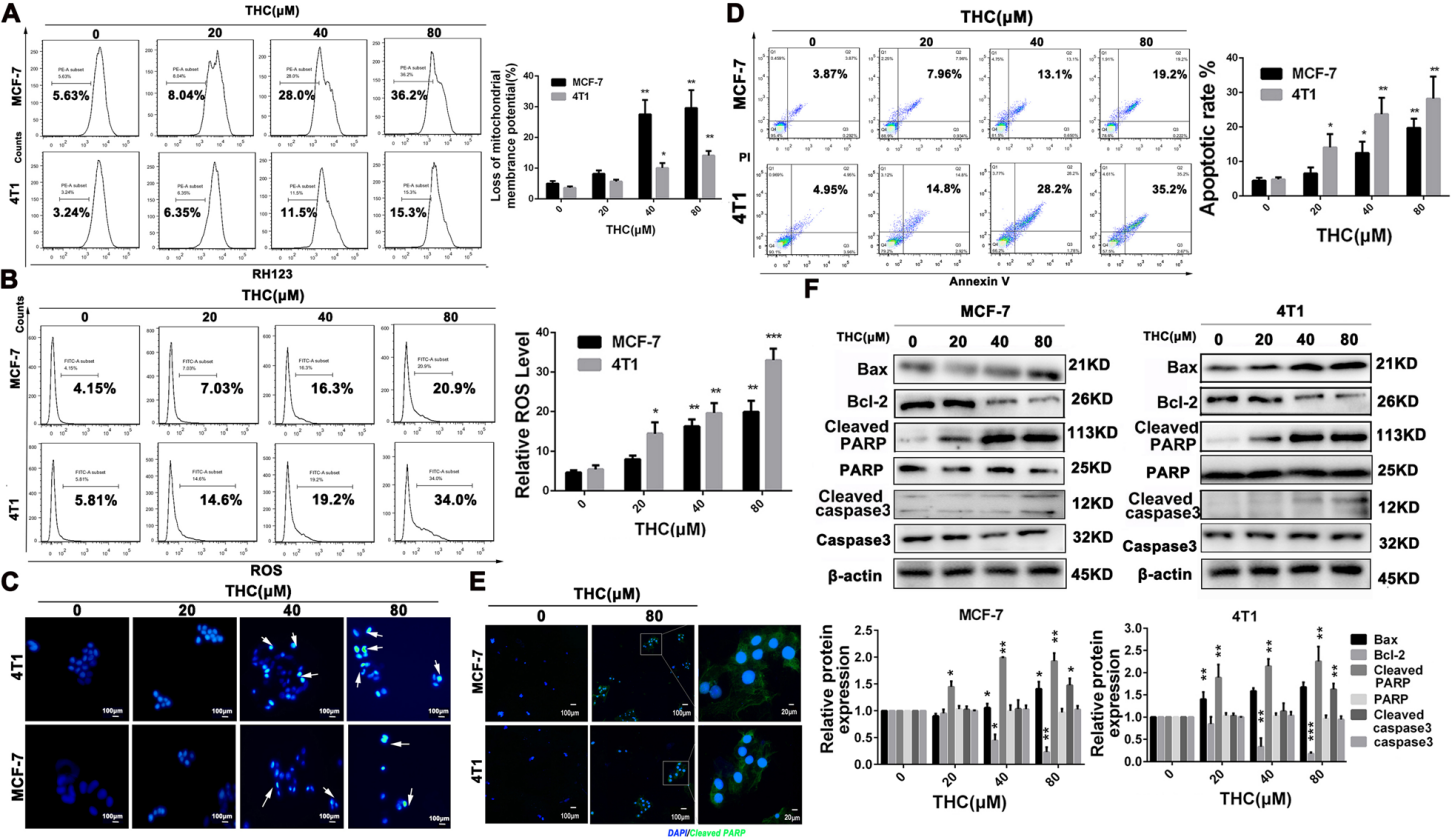
Effects of THC on apoptosis of breast cancer cells. **A** THC changed the mitochondrial membrane potential (ΔΨm) of breast cancer cells. MCF-7 and 4T1 cells were treated with various concentrations (0–80 μM) of THC for 48 h and then detect the change in ΔΨm by FCM. Data are expressed as means ± SD from three experiments (*P < 0.05; **P < 0.01; ***P < 0.001 compared to control). **B** MCF-7 and 4T1 cells were treated with designated concentrations (0–80 μM) of THC for 48 h. The harvested cells were loaded with DCFH-DA and then ROS levels in the cells were measured by FCM. Data are expressed as means ± SD from three experiments (*P < 0.05; **P < 0.01; ***P < 0.001compared to control). **C** Cell nuclear changes of MCF-7 and 4T1 cells were determined by staining with Hoechst 33258 and visualized by a fluorescence microscope after treatment with increasing doses of THC (0–80 μM) for 48 h. **D** MCF-7 and 4T1 cells were treated with THC (0–80 μM) at indicated doses for 48 h, and the level of apoptosis was evaluated using the Annexin V/PI dual-labeling technique,and determined by FCM. Data are expressed as means ± SD from three experiments (*P < 0.05; **P < 0.01; ***P < 0.001 compared to control). **E** The effect of THC (0, 80 μM) on Cleaved PARP protein expression was analyzed by immunofluorescence. F: Western blot analyses of MCF-7 and 4T1 cells treated (48 h) with different concentrations (0–80 μM) of THC were used to evaluate protein expression of Bax and Bcl-c, caspase-3, Cleaved capase-3, PARP and Cleaved PARP. Protein expression was quantified by the densitometry analysis using Image J and normalized against β-actin expression. Data are expressed as means ± SD from three experiments (*P < 0.05; **P < 0.01; ***P < 0.001 compared to control)

We used mitochondria-specific and voltage-dependent dye Rh123 to detect alterations in ΔΨm in MCF-7 and 4T1 cells and determine whether THC induces the disruption of the mitochondrial membrane potential. As shown in Fig. 2A, B, THC treatment led to the loss of ΔΨm potential in MCF-7 and 4T1 cells. These results suggest that the mitochondria-mediated pathway may be involved in THC-induced apoptosis. The ROS accumulation can change the intracellular environment and induce apoptosis. For the detection of ROS, we stained the cells with DCFH-DA reagent and estimated ROS accumulation by FCM. We discovered that the treatment of THC lessened ROS accumulation in a dose-dependent manner, which can be observed in Fig. 2C explicitly. Cell apoptosis was assessed by annexin V-FITC/PI staining. The results show that a high percentage of cell death and apoptosis was found in MCF-7 and 4T1 cells after THC treatment (Fig. 2D). Immunofluorescence assay showed that THC (80 μM) could enhance the expression level of cleaved PARP in cells (Fig. 2E). Cell apoptosis–related proteins were detected by WB assay, and the experimental results showed (Fig. 2F) that THC increased the expression level of cleaved PARP and cleaved caspase-3 in MCF-7 and 4T1 cells, and ratio of Bax/Bcl2 was also elevated in both MCF-7 and 4T1 cells.

### THC inhibits metastasis of breast cancer cells by affecting epithelial–mesenchymal transition (EMT) transformation

**Fig. 3:**
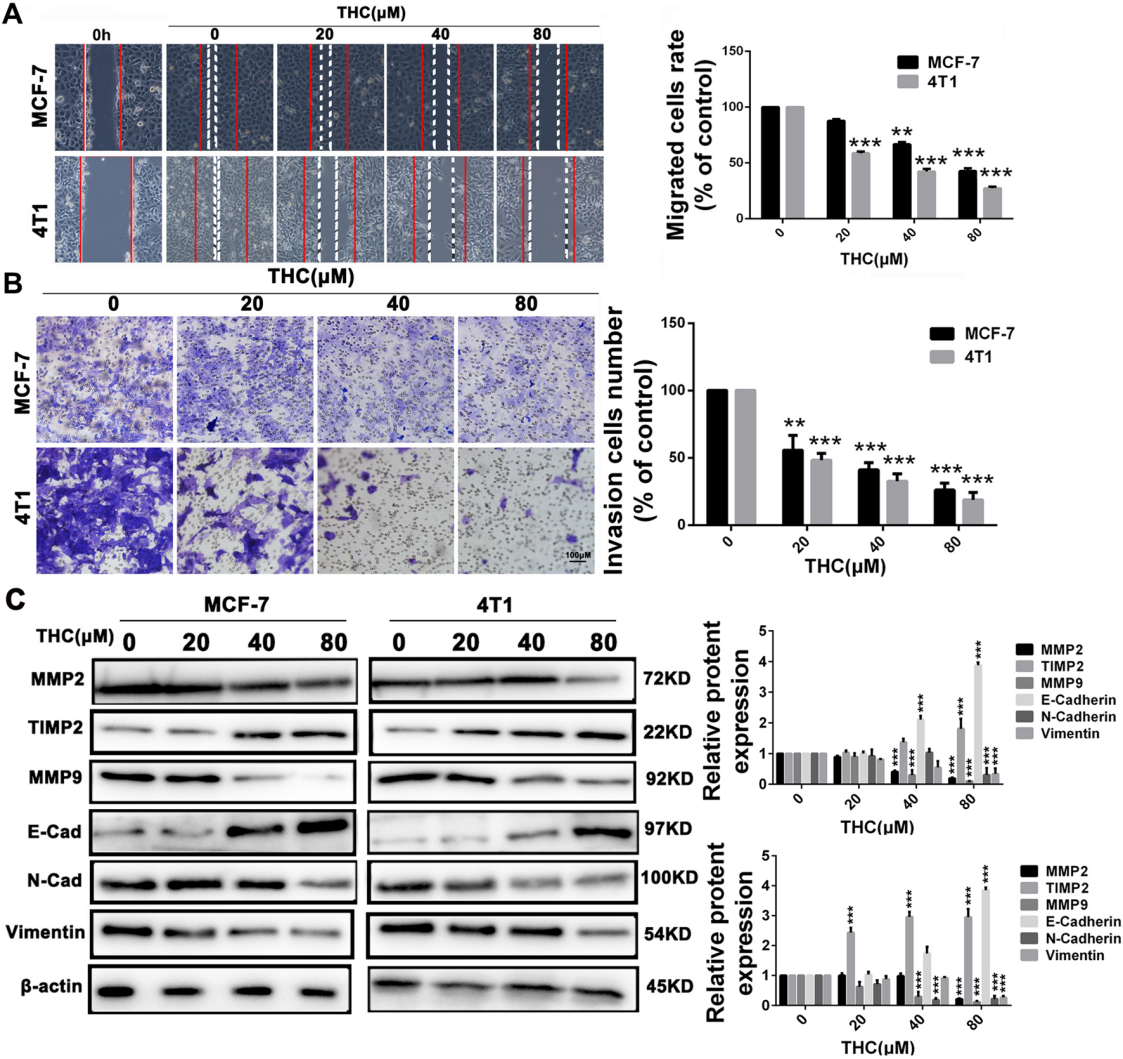
Inhibition of migratory and invasive in MCF-7 and 4T1 cells by THC treated. **A** MCF-7 and 4T1 cells were seeded on six-well plates. A single scratch was made after the cells grew about 90% confluence. After treatment of THC for 48 h, the cells were fixed and photographed. The lines indicate the area occupied by the initial scraping and migrated cells were quantified. **B** MCF-7 and 4T1 cells were seeded in the top chamber of transwell with serum-free medium and treated with vehicle or different concentrations of THC. After about 48 h, migrated cells were fixed, stained, photographed and quantified. **C** MCF-7 and 4T1 cells were treated with different doses of THC for 48 h, cells were harvested and western blot assay was carried out to detect the expression of MMP2, MMP9, TIMP2, E-cadherin, N-cadherin, Vimentin and β-actin served as loading control. Protein expression of MMP-2, MMP-9 and TIMP2 were quantified by the densitometry analysis using Image-Pro Plus and normalized against β-actin expression. Data are expressed as means ± SD from three experiments. (**P < 0.01, ***P < 0.001, compared to control)

We performed wound-healing and Transwell migration assay to assess anti-migration effect of THC in MCF-7 and 4T1 cell lines. As shown in Fig. 3A, THC inhibits the migration of MCF-7 and 4T1 cells in a dose-dependent manner. Then, we performed Matrigel invasion assay. The invasion ability of MCF-7 and 4T1 cells significantly decreased in the presence of THC over that of the vehicle, which can be explicitly observed in Fig. 3B. Furthermore, considering the relationship among EMT transformation, metalloproteinase, and cell migration and invasion, we investigated whether metalloproteinases and EMT transformation are involved in an inhibitory effect on the migration and invasion of THC. As shown in Fig. 3C, THC treatment decreased the expression level of MMP-9 and MMP-2 in breast cancer cells and increased the expression level of TIMP2. In addition, THC treatment decreased the expression level of N-Cadherin and vimentin in breast cancer cells and increased the expression level of E-Cadherin. Collectively, these results indicate that THC inhibited the migration and invasion of breast cancer cells.

### Transcriptomic analysis and molecular docking of THC in breast cancer cells

**Fig. 4:**
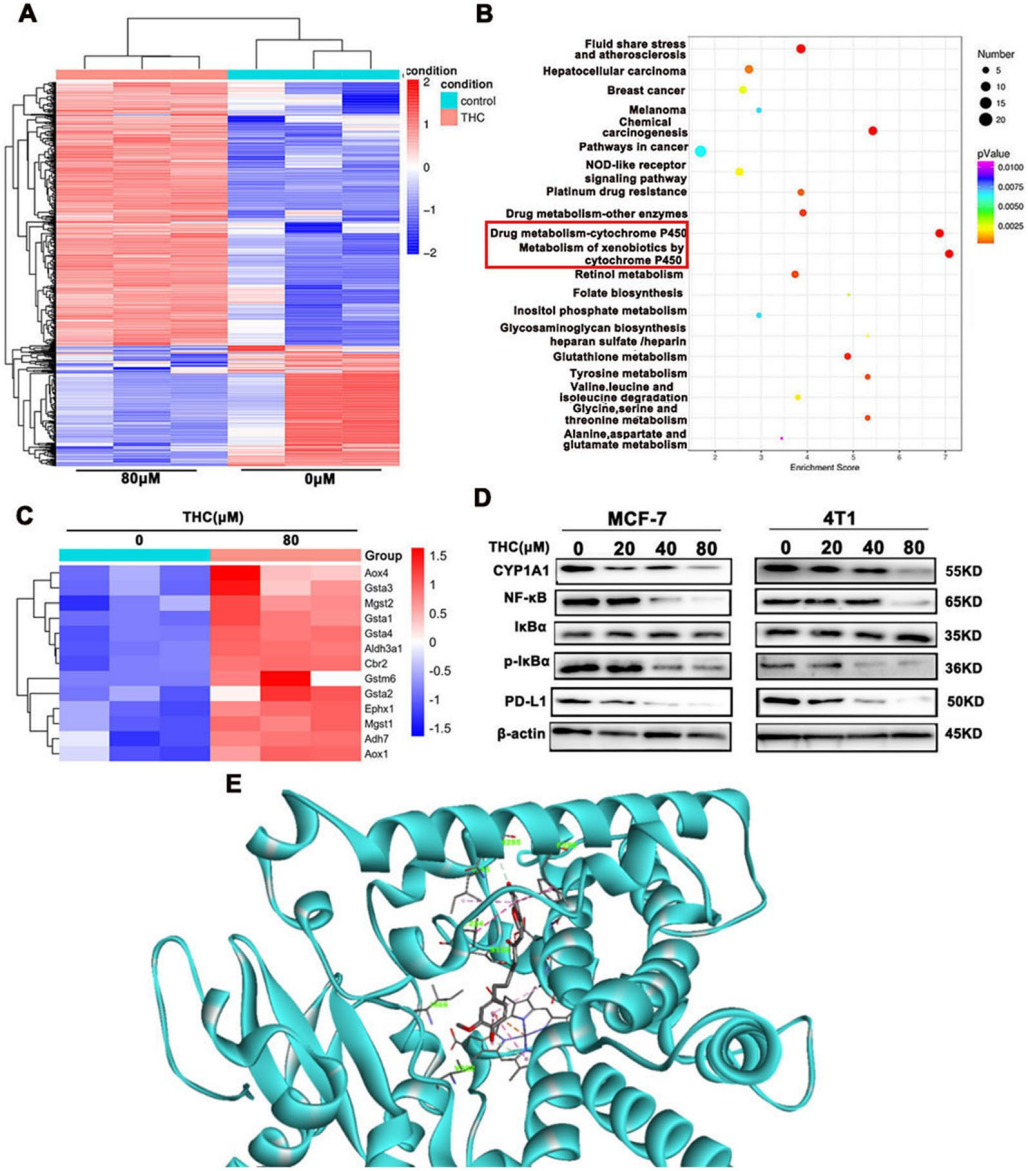
Transcriptomic analysis and molecular docking of THC in breast cancer cells. **A** Heatmap depiction of differentially expressed genes between different treated groups in 4T1 cells. **B** KEGG analysis of representative signaling pathways enrichment between different treated groups in 4T1. **C** Heatmap depiction of differentially expressed genes of Cytochrome P450 enzyme metabolic system signaling pathways. **D** After different concentrations of THC were stimulated, the CYP1A1, NF-κb, iκBa, phosphorylated iκBa and PD-L1 protein expression was verified by Western Blot assay in MCF-7 and 4T1cells. E: Molecular binding mode of THC and P4501A1 protein

THC could significantly impair viability and induce apoptosis in 4T1 and MCF-7 cells. We utilized transcriptomics data to study the effects of THC on 4T1 cells and gain insight into the molecular mechanism of the selective function of THC. As shown in Fig. 4A, after treatment with THC, changes in the transcriptome with 4T1 cells were distinctly different from those in untreated 4T1 cells. Subsequently, we performed Kyoto Encyclopedia of Genes and Genomes analysis of the signaling pathway to determine the molecular pathway associated with THC (Fig. 4B). THC stimulation primarily affects the CYP450 metabolic pathway. Differentially expressed genes in this pathway are shown in Fig. 4C. Through analysis and research, the CYP1A1 protein was found to be highly expressed in various breast cancer and gynecological tumors, and these differential genes may be involved in the NF-κB signaling pathway and the regulation of tumor immunity. In verifying the results of the transcriptomic experiment, THC-treated breast cancer cells were detected by WB assay, and CYP1A1, PD-L1, and NF-κB signaling pathway-related proteins were detected. The results showed (Fig. 4D) that THC inhibited the expression of CYP1A1, NF-κB, P-IκBα, and PD-L1 in breast cancer cells.

Molecular docking results (Fig. 4E) showed that THC had a binding pattern similar to that of other known P4501A1 ligands. One end of the aromatic ring of THC binds to the hydrophobic cavity formed by many hydrophobic residues such as A317, V382 and I386 around the porphyrin ring and forms π–π stacking with the P4501A1 porphyrin ring. The THC carbonyl oxygen forms a hydrogen bond with the hydroxyl hydrogen of S122. The aromatic ring structure at the other end of THC is well embedded between F224 and F258 at the top of the catalytic domain of P4501A1 to form a good π–π stacking effect. It indicates that THC has strong binding ability with CYP1A1.

### Regulation of THC on the NF-κB signaling pathway and PD-L1 protein expression after overexpression of the CYP1A1 gene

**Fig. 5:**
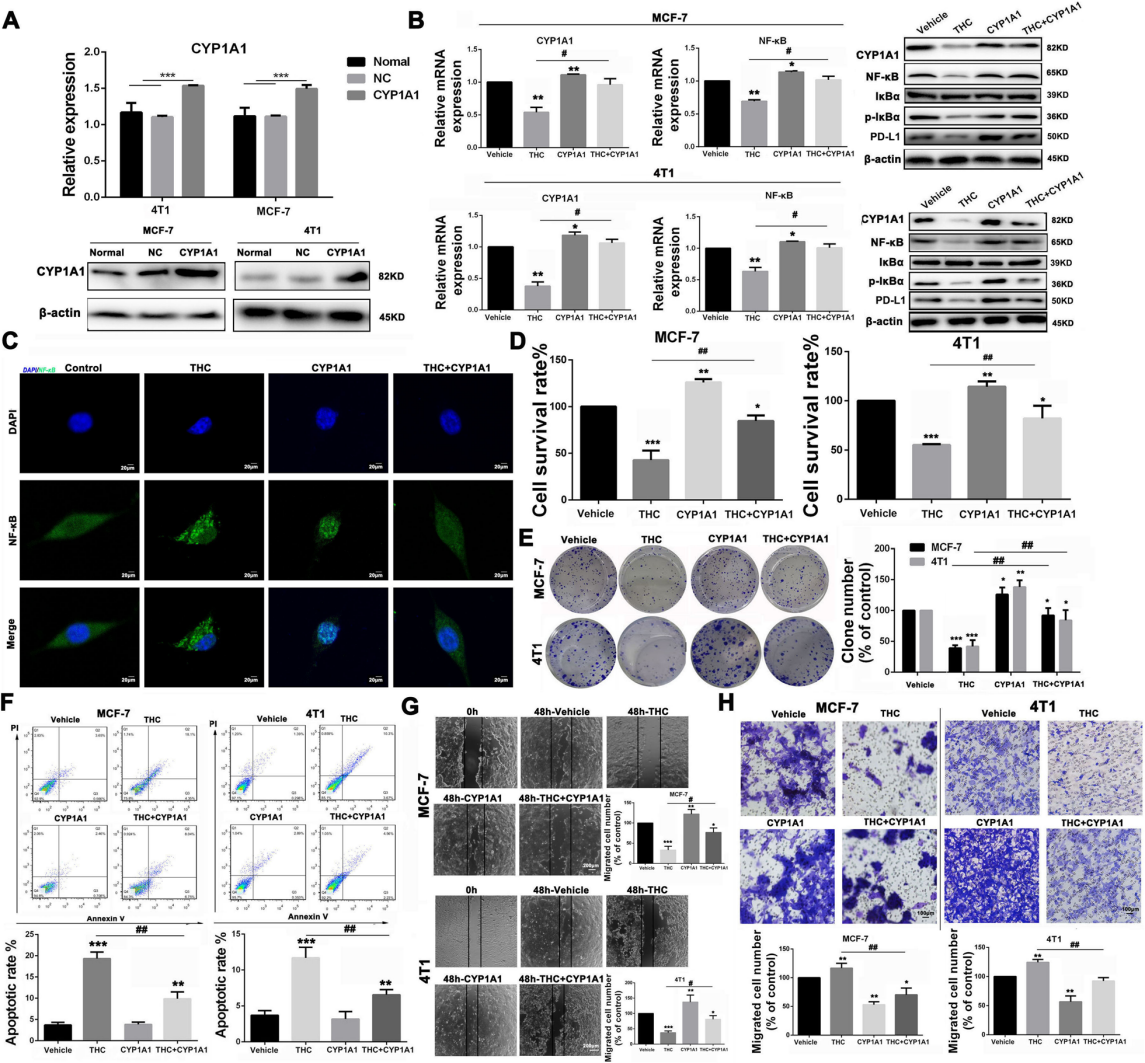
Effects of CYP1A1 overexpression on THC anti-breast cancer. **A** After synthesis of the overexpressed plasmid CYP1A1, MCF-7 and 4T1 cancer cells were transfected with Lipo2000. Changes of mRNA expression levels of CYP1A1 in MCF-7 and 4T1 were mesasured. **B** The MCF-7 and 4T1 cancer cells were divided into four groups: Vehicle, THC (80 μM), CYP1A1 and CYP1A1 + THC (80 μM). The expression of CYP1A1, NF-κB associated protein and PD-L1 in each group were detected by RT–PCR and WB experiments. **C** Immunofluorescence analysis of nuclear transportation of NF-κB protein in MDA-MB-231 cells. **D** CCk8 assay was used to analyze the effect of transfection of overexpressed plasmid on the cytotoxicity of MCF-7 and 4T1 cancer cells. **E** The effect of transfection of overexpressed plasmids on the clonogenesis of breast cancer cells was analyzed. **F** The effect of THC on apoptosis of breast cancer cells transfected with overexpressed plasmid was using the Annexin V/PI dual-labeling technique, and determined by FCM. **G** The effect of THC on breast cancer cell metastasis after transfection with overexpressed plasmid was analyzed by scratch assay. **H** Transwell assay was used to analyze the effect of THC on invasion of breast cancer cells after transfection with overexpressed plasmid. Data are expressed as means ± SD from three experiments. (*P < 0.05; **P < 0.01; ***P < 0.001, compared to control; ^#^P < 0.05; ^##^P < 0.01; ^###^P < 0.001, compared to THC)

MCF-7 and 4T1 cells were transfected by constructing a CYP1A1 overexpression plasmid. The experiment was divided into four groups: the solvent control group (vehicle group: normal breast cancer cells + solvent), THC stimulation group (THC group: normal breast cancer cells + THC 80 μM), CYP1A1 overexpression group (CYP1A1 group: overexpression of CYP1A1 breast cancer cells), and THC-stimulated overexpression of the CYP1A1 breast cancer cell group (THC + CYP1A1 group: THC 80 μM + overexpression of CYP1A1 breast cancer cells). The results showed that after transfection of CYP1A1 plasmid, the expression level of CYP1A1 increased in cells. The transfected breast cancer cells were stimulated with THC (80 μM) for 48 h, and WB assay showed (Fig. 5A) that NF-κB and p-IκBα expression increased in the CYP1A1 group compared with the THC group. By contrast, the expression level of NF-κB and p-IκBα in the CYP1A1 group was decreased compared with the THC + CYP1A1 group. Meanwhile, the experimental results showed (Fig. 5B) that the survival rate of breast cancer cells increased after the overexpression of CYP1A1 compared with the blank control group, and the inhibitory effect of THC on the proliferation of breast cancer cell was weakened after the overexpression of CYP1A1. In clone formation experiment (Fig. 5C), the formation speed and size of breast cancer cell clones in the CYP1A1 group increased compared with the control group, whereas the formation ability of breast cancer cell clones in the THC + CYP1A1 group decreased compared with the THC group. Meanwhile, after the overexpression of the CYP1A1 gene in breast cancer cells, the experimental results (Fig. 5D) show that the cells in the THC group undergo apoptosis, whereas the breast cancer cells in the CYP1A1 group are in a good state with almost no apoptosis. In addition, the breast cancer cells in the THC + CYP1A1 group undergo almost no apoptosis. These results indicate that CYP1A1 overexpression affects the regulation of apoptosis in breast cancer cells with high doses of THC. Moreover, THC can inhibit the metastasis of breast cancer cells in vitro by inhibiting EMT transformation. After the overexpression of the CYP1A1 gene, experimental results showed that the inhibitory effect of high-dose THC on metastasis of breast cancer cells was significantly weakened. As shown in Fig. 5E, F, the “wound recovery” and invasion ability of breast cancer cells in the CYP1A1 group were enhanced, whereas the metastasis and invasion ability of cells in the THC + CYP1A1 group were enhanced compared with that in the THC group. Furthermore, THC on the NF-κB signaling pathway and PD-L1 protein expression were regulated after overexpression of the CYP1A1 gene.

### Knockdown of the CYP1A1 gene and regulation of the NF-κB signaling pathway and PD-L1 protein expression using THC

**Fig. 6:**
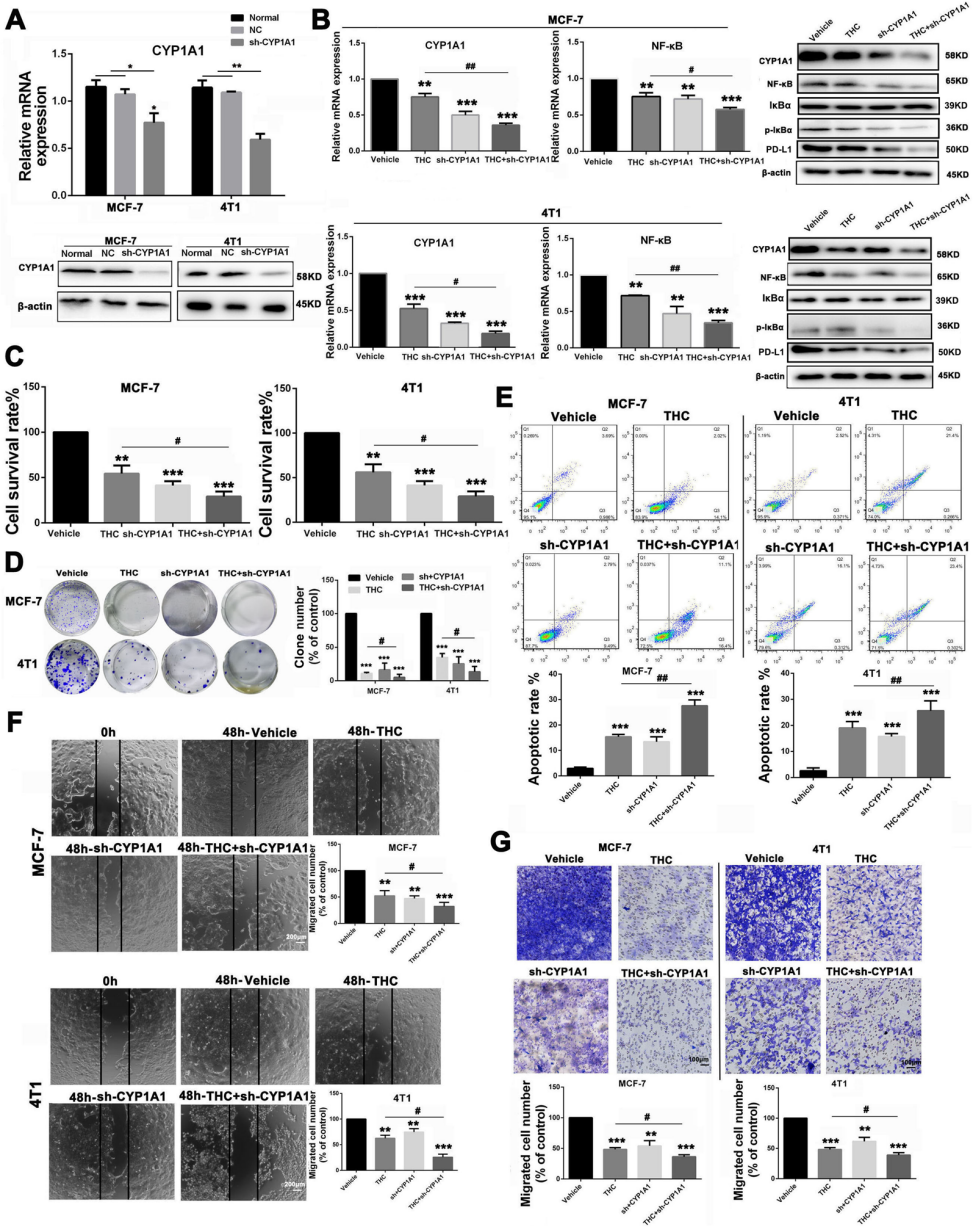
Effects of CYP1A1 knockdown on THC anti-breast cancer. **A** After synthesis of CYP1A1 knock-down plasmid, MCF-7 and 4T1 cancer cells were transfected with Lipo2000. Changes of mRNA expression levels of CYP1A1 in MCF-7 and 4T1 were mesasured. **B** The MCF-7 and 4T1 cancer cells were divided into four groups: Vehicle, THC (80 μM, sh-CYP1A1 and sh-CYP1A1 + THC(80 μM). The expression of CYP1A1, NF-κB associated protein and PD-L1 in each group were measured by RT–PCR and WB experiments. **C** CCK8 assay was used to analyze the effect of transfection of knock-down plasmid on the cytotoxicity of MCF-7 and 4T1 cancer cells. **D** The effect of transfection of knock-down plasmid on the clonogenesis of breast cancer cells was analyzed. **E** The effect of THC on apoptosis of breast cancer cells transfected with knock-down plasmid was using the Annexin V/PI dual-labeling technique, and determined by FCM. **F** The effect of THC on breast cancer cell metastasis after transfection with knock-down plasmid was analyzed by scratch assay. **G** Transwell assay was used to analyze the effect of THC on invasion of breast cancer cells after transfection with knock-down plasmid. Data are expressed as means ± SD from three experiments. (*P < 0.05; **P < 0.01; ***P < 0.001, compared to control; ^#^P < 0.05, ^##^P < 0.01, compared to THC)

MCF-7 and 4T1 cells were transfected by constructing the CYP1A1 knockdown plasmid. After transfection, the expression of CYP1A1 in cells was detected by reverse transcription polymerase chain reaction (RT–PCR) and WB assay. The experiment was divided into four groups: solvent control group (vehicle group: normal breast cancer cells + solvent), high-dose THC stimulation group (THC group: normal breast cancer cells + THC 80 μM), CYP1A1 knockdown group (sh-CYP1A1group: CYP1A1 knockdown group), and THC-stimulated knockdown group (THC + sh-CYP1A1 group: THC 80 μM + sh-CYP1A1 breast cancer cells). The transfected breast cancer cells were stimulated with THC (80 μM), and WB experiments were performed 48 h after stimulation. The results showed that the expression level of NF-kB and p-IkBa was decreased in the THC, sh-CYP1A1, and THC + sh-CYP1A1 groups (Fig. 6A). Compared with the sh-CYP1A1 group, the expression level of NF-κB and p-IκBα in the THC + sh-CYP1A1 group also decreased. After CYP1A1 knockdown, group (Fig. 6B), and the inhibitory effect of THC on the proliferation of breast cancer cells was enhanced after CYP1A1 knockdown. In the clone formation experiment (Fig. 6C), the formation rate of breast cancer cells in the sh-CYP1A1 group decreased compared with the vehicle and size of breast cancer cell clones in the sh-CYP1A1 group were lower than those in the vehicle group, and the formation ability of breast cancer cell clones in the THC + sh-CYP1A1 group was lower than that in the high-dose THC group. These results indicate that the knockdown of CYP1A1 can positively regulate the anti-proliferative effect of THC on breast cancer. Based on the experimental results (Fig. 6D), after CYP1A1 gene knockdown in breast cancer cells and administration with 80 μM of THC, breast cancer cells experienced significant apoptosis. After CYP1A1 knockdown, the apoptosis rate of breast cancer cells in the sh-CYP1A1 group increased. In addition, after CYP1A1 knockdown and THC stimulation, we found that the apoptosis rate of breast cancer cells in the THC + sh-CYP1A1 group was significantly higher than that in the sh-CYP1A1 group, indicating that CYP1A1 knockdown enhances the role of THC in promoting the apoptosis of breast cancer cells. After knockdown of the CYP1A1 gene, experimental results showed (Fig. 6E, F) that the metastasis effect of breast cancer cells in the THC group was significantly inhibited compared with the solvent group, whereas the “wound recovery” ability and invasion ability of breast cancer cells in the sh-CYP1A1 group were also weakened. Moreover, compared with the sh-CYP1A1 group, the metastasis and invasion ability of the THC + sh-CYP1A1 group were significantly reduced. These results suggest that the knockdown of CYP1A1 in breast cancer cells can promote the inhibitory effect of THC on breast cancer metastasis.

### THC inhibits tumor growth in vivo through the CYP1A1/NF-κB axis in MCF-7-bearing nude mice

**Fig. 7:**
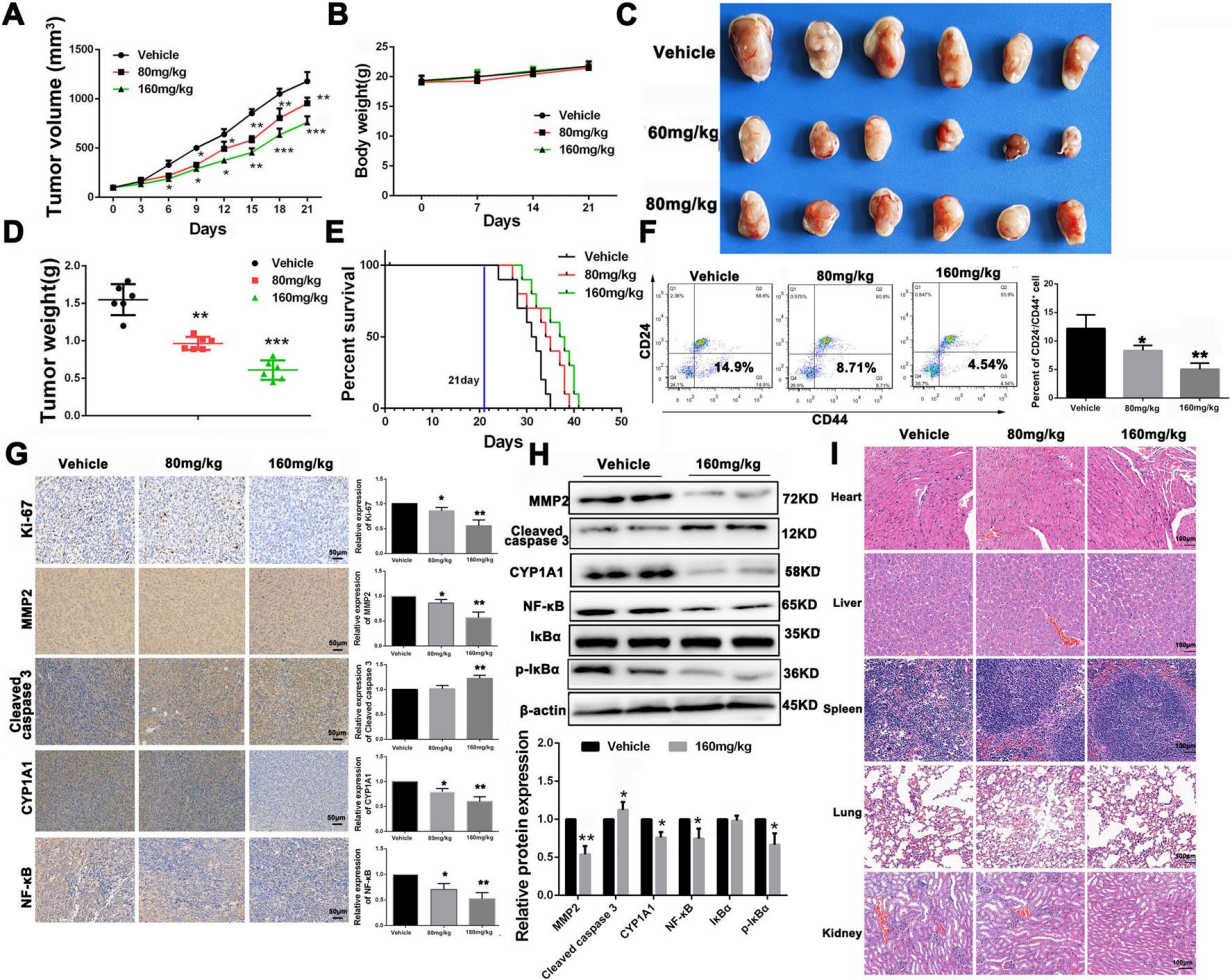
Effects of THC on tumor progression in MCF-7 tumor-bearing mice. **A** The MCF-7 tumor growth curves of different groups within the treatment process. **B** Variation of mice body weight of different groups within treatment progress. **C** Representative image of MCF-7 tumors of different groups at the termination of the experiment. **D** MCF-7 tumor weight of different treated groups at the termination of experiment. **E** Survival time of MCF-7 tumor-bearing mice of different treated groups at the termination of experiment. **F** The number of tumor stem cell-like cells (CD44+ CD24−) in tumor tissues of mice after THC administration. **G** Immunohistochemistry analysis of Ki-67, MMP-2, Cleaved caspase-3 and CYP1A1 of MCF-7 tumor sections after different treatments. **H** Expression levels of MMP-2, Cleaved caspase-3 and CYP1A1, NF-κB, IκBα and p-IκBα associated proteins of different treated MCF-7 tumor tissues were detected by western blot. I: H&E staining was used to analyze the pathological changes of the heart, liver, spleen, lung and kidney in each group after THC administration. Bars represent means ± SD of at least 3 independent experiments; *P < 0.05, **P < 0.01 and ***P < 0.001 compared with the Vehicle group

MCF-7 tumor-bearing mice were treated with THC at 80 and 160 mg/kg to determine whether the in vivo antitumor efficiency of THC was consistent with its effects in vitro. As shown in Fig. 7A and C, D, treatment with THC at 80 or 160 mg/kg significantly inhibited MCF-7 tumor growth and weight in a dose-dependent manner compared with the vehicle group. After analyzing the number of tumor stem cell–like cells in tumor tissue (Fig. 7F), THC can reduce the number of cells (CD44^+^/CD24^−^). Moreover, compared with the vehicle group, the survival rate of tumor-bearing mice was significantly prolonged after treatment with various concentrations of THC (Fig. 7E). The body weight and important viscera of mice did not present any abnormal variation during the treatment process (Fig. 7B and I). Furthermore, the results of immunohistochemistry analysis of tumors (Fig. 7G) indicated that THC inhibited the proliferation of nuclear Ki-67-positive cells, enhanced the expression of cleaved caspase-3 to induce cell apoptosis, reduced the expression of MMP-2 to inhibit metastasis, and downregulated the expression levels of NF-κB in tumor sections. Moreover, WB analysis of isolated tumors revealed that THC reduced the expression level of CYP1A1, inhibited the nuclear expression level of NF-κB, and downregulated the expression level of p-IκBα (Fig. 7H). Overall, the abovementioned data suggest that THC could suppress MCF-7 cell growth by inhibiting the CYP1A1/NF-κB signaling pathway, which is consistent with in vitro data.

### THC regulates the TIME through the CYP1A1/NF-κB/PD-L1 axis to inhibit tumor growth in 4T1-bearing mice

**Fig. 8:**
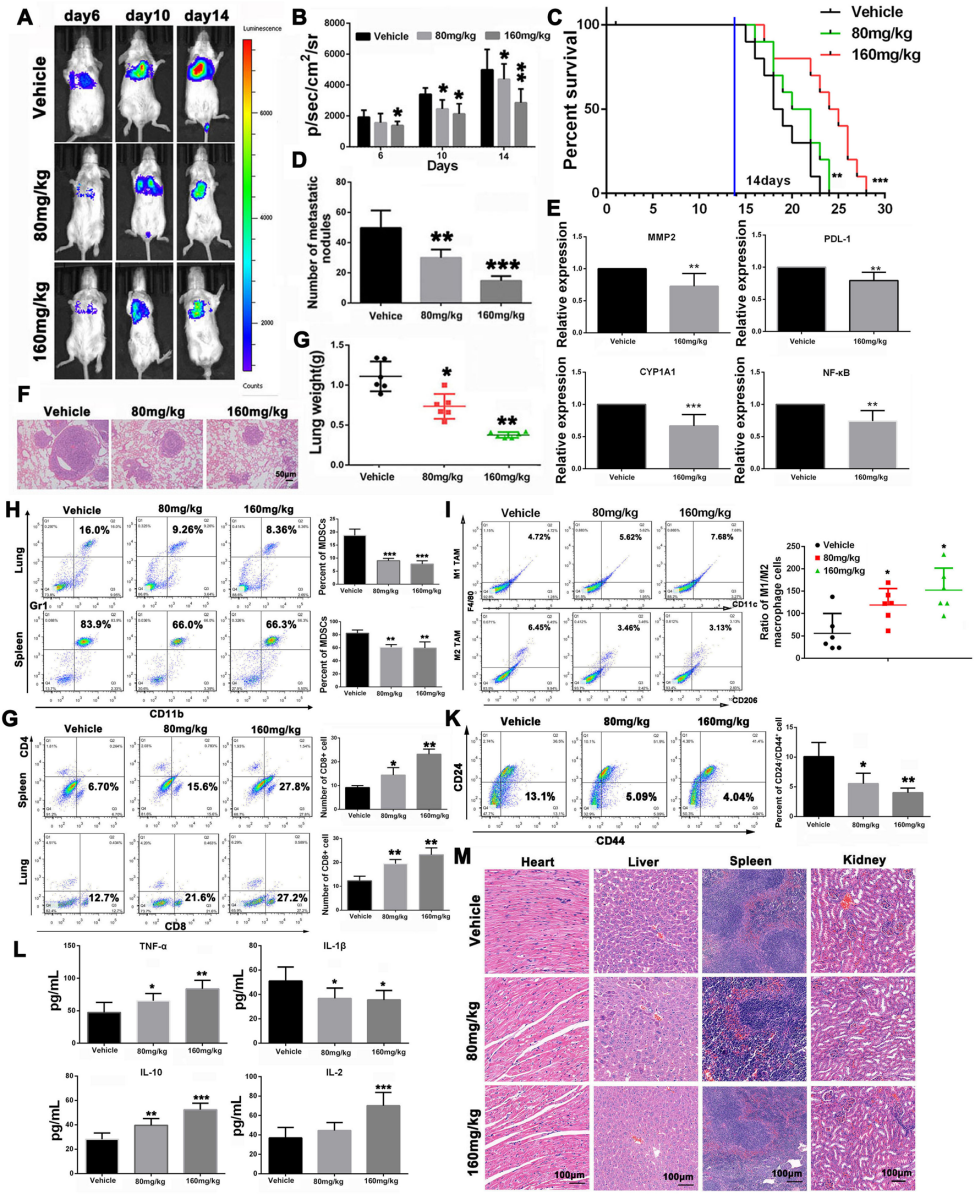
THC regulates tumor immune microenvironment by CYP1A1/NF-κB/PD-L1 axis to inhibit tumor growth in 4T1-bearing mice. **A** Bioluminescence images of mice bearing a pulmonary metastasis model of 4T1-luciferase cells after different treatments at determined time points (6, 10, or 14 days). **B** Quantitation of the bioluminescence signal in mice after different treatments at determined time points. **C** Survival time of 4T1-bearing mice after different treatments. **D** Number of nodules of lungs from different treated groups. **E** Lung tissues of lung metastases mice were taken for PCR test to detect the changes of MMP2, NF-κB, CYP1A1 and PD-L1 genes. **F** H&E staining analysis of lung tissues from different treated groups. **G** Lung weight of the 4T1-bearing mice after different treatments. **H** Changes of proportion of MDSCs in 4T1 tumors after different treatments. **I** Effect of THC on the number of M1 and M2 macrophages in tumor tissue. **J** Changes of proportion of CD4 + and CD8 + T cells in 4T1 tumors after different treatments. **K** The number of tumor stem cell-like cells (CD44+ CD24−) in tumor tissues of mice after THC administration. **L** Detection of tumor immune-related cytokines. **M** H&E staining was used to analyze the pathological changes of the heart, liver, spleen, lung and kidney in each group after THC administration. Bars represent means ± SD of at least 3 independent experiments; *P < 0.05, **P < 0.01 and ***P < 0.001 compared with the Vehicle group

Given the anti-metastasis effect of THC in vitro, we subsequently established a pulmonary metastasis model of 4T1-luciferase cells in BALB/c mice to evaluate the anti-metastatic efficacy of THC in vivo. As shown in Fig. 8A, B, D, and G, the bioluminescence representing metastatic tumor cells was evidently enhanced over time compared with the THC treatment group. At 15 days after tumor inoculation, the bioluminescence signals of mice in the 80 and 160 mg/kg treatment groups were significantly lower than those of the vehicle group. In addition, compared with the vehicle group, the survival rate of pulmonary metastatic mice was significantly prolonged after treatment with various THC concentrations (Fig. 8C). Moreover, the number of metastatic nodules at lung sites in the 80 and 160 mg/kg treatment groups was significantly reduced compared with the vehicle group. Furthermore, the collected lung tissue was sectioned and analyzed by H&E staining to determine the anti-metastatic efficiency of THC (Fig. 8F). H&E staining of these tissue sections indicated that fewer tumor nodules were present in the THC treatment group compared with the vehicle group. These results indicated that THC could effectively inhibit 4T1 tumor pulmonary metastases. After THC (160 mg/kg) was administered, the lung tissue of lung metastatic mice was collected for PCR test to detect the changes in MMP2, NF-κB, CYP1A1, and PD-L1 genes in tissues (Fig. 8E). The experimental results showed (Fig. 8H) that THC could decrease the number of MDSC in lung and spleen tissues of tumor-bearing mice (**P < 0.01; ***P < 0.001), reduce immunosuppression, enhance immune response, and play an antitumor effect. After THC treatment, tumor tissues of mice in each group were labeled with antibodies (F4/80, CD206, and CD11c), and the polarization of M1/M2 was observed by FCM. The results showed that THC could increase the proportion of M1-type macrophages in lung tissue (Fig. 8I, *P < 0.05). The experimental results showed that THC had no effect on the number of CD4 + T lymphocytes (Fig. 8J), but it could increase the number of CD8 + T lymphocytes in mice. The number of MDSCs (Gr-1 and CD11b labeled) in tumor and spleen tissues of mice in each group was detected by FCM to determine the mechanism of THC tumor induction and regulation of tumor microenvironment in vivo. After THC was administered, the number of stem cell–like cells in lung metastatic tumor tissues of mice was detected, which could demonstrate the inhibitory effect of THC on tumor growth. The tumor tissue was digested into single cells and labeled with CD24 and CD44 antibodies. The number of CD44^+^/CD24^−^ cells was measured by FCM. The results showed (Fig. 8K) that THC reduced the number of stem cell–like cells in lung metastatic tissue (**P < 0.01; ***P < 0.001), thereby inhibiting lung metastasis of breast cancer. THC decreased the level of interleukin-1β (IL-1β) in the peripheral blood of mice with lung metastasis (Fig. 8L, *P < 0.05) and increased the content of tumor necrosis factor (TNF-α), interleukin 2 (IL-2), and interleukin 10 (IL-10; *P < 0.05; **P < 0.01; ***P < 0.001). Through pathological examination, the heart, liver, spleen, and kidney of nude mice were observed (Fig. 8M), and no evident lesions were found. THC has low toxicity and safety.

## Discussion and conclusions

THC has a wide range of pharmacological activities, and its antitumor activity has been widely reported [19]. In this study, CCK8, clonogenesis assay, EDU assay, and cell cycle analysis confirmed that THC could inhibit the activity, affect the cell cycle, and inhibit the proliferation of breast cancer cells. In addition, in vivo models of MCF-7 cells and 4T1 cell xenograft tumor mice indicated that THC can inhibit the growth of breast cancer cells, delay the tumor growth rate, reduce the expression of Ki-67 in tumor tissues, and increase the survival time of tumor-bearing mice. The mitochondria are the main source of ROS production, and ROS play an important role in cell apoptosis by altering mitochondrial membrane potential (ΔΨm) [16]. Anti-apoptotic protein Bcl-2 and pro-apoptotic protein Bax are involved in the mitochondria-mediated apoptotic pathway [15]. In addition, studies have shown that THC can induce apoptosis of H22 liver ascites tumor cells through the mitochondrial pathway, reduce the expression of Bcl-2/Bax protein, and increase the expression level of p53 protein [10]. Yoysungnoen B. et al.[20]. also found that THC can promote tumor cell apoptosis through the ERK signaling pathway in a cervical cancer xenotransplantation model. THC has been reported to activate caspase-3 and caspase-9 proteins through the mitochondrial pathway of cell apoptosis when promoting apoptosis of MCF-7 cells in vitro [21]. Therefore, these results suggest that THC therapy triggers breast cancer cell apoptosis through the ROS-mediated mitochondrial apoptotic pathway. In this study, Hochest33258 staining, FCM, and WB detection showed that after THC treatment, apoptotic bodies were generated; mitochondrial membrane potential was changed; intracellular ROS content was increased, and Bax/Bcl-2 protein expression was increased [22]. Cleaved caspase-3 protein was activated. Cleaved caspase-3 expression was increased, and Cleaved caspase-3 protein expression was increased in mouse tumor tissues after THC administration by immunohistochemistry and WB assay in in vivo animal experiments. These results indicate that THC can induce the apoptosis of breast cancer cells through the mitochondria-mediated apoptotic pathway. This transformation is abnormally activated in tumor cells, leading to tumor invasion and trans. Studies have shown that metastasis is the primary cause of death in 90% of malignant tumors. Therefore, EMT transformation plays an important role in the occurrence and development of tumors. In this study, we used scratch assay, Transwell invasion assay, and WB assay to evaluate the anti-metastasis effect of THC in MCF-7 and 4T1 cells. Scratch assay and Transwell assay showed that THC could significantly inhibit the migration and invasion of breast cancer cells in vitro, inhibit EMT transformation of breast cancer cells, and reduce the expression of MMP2. Our in vivo study also showed that THC could significantly inhibit lung metastasis in 4T1-bearing mice, reduce the expression of MMP2 in tumor tissues, and prolong the survival time. Based on previous reports, CYP1A1 is overexpressed in a variety of malignant tumors, and it has a certain correlation with the incidence of breast cancer, cervical cancer [23]. In addition, the high expression of CYP1A1 can induce the occurrence of a variety of malignant tumors. Clinical studies have shown that the high expression of CYP1A1 is correlated with the malignant degree and prognosis of breast cancer. Thus, the inhibition of CYP1A1 may be a potential therapeutic target for the treatment of breast cancer [12]. The NF-κB signaling pathway is closely related to tumor genesis and development. Abnormal NF-κB pathway activation directly or indirectly affects SNAIL, SLUG, and ZEB1 expression levels [24, 25]. Moreover, IκBα inhibits NF-κB activation, and it can be phosphorylated by the activated Iκκ complex (NEMO, Iκα, and Iκβ) to induce self-degradation. Subsequently, the NF-κB transcription complex is released into the nucleus, where it subsequently binds to negative regulatory DNA elements and promotes the transcription of pre-transfer genes [25]. Studies have shown that the activation of NF-κB and AP-1 signaling pathways is directly involved in the regulation of CYP1A1 by heavy metals. Furthermore, nicotine stimulates CYP1A1 expression through transcription factor, activator protein 1, NF-κB, and aromatic hydrocarbon receptor signaling pathways [26]. In this study, transcriptional analysis revealed significant changes in CYP1A1 after THC treatment in breast cancer cells. WB and RT–PCR experiments verified that THC treatment decreased the expression level of CYP1A1, NF-κB, and p-IκBα. Molecular docking showed that THC had a good binding ability with the CYP1A1 protein. The expression levels of NF-κB and p-IκBα were increased after transfection of breast cancer cells MCF-7 and 4T1 with CYP1A1 overexpression. However, after CYP1A1 knockdown and THC stimulation, the expression levels of NF-κB and p-IκBα in MCF-7 and 4T1 cells decreased remarkably. Therefore, THC can affect NF-κB expression through CYP1A1.

Inflammation is associated with immunity, and controlling the inflammatory process is a main activity of NF-κB protein. TNF-α is also a widely studied pro-tumor cytokine, and its enhanced expression in many cancers is often associated with poor prognosis [25]. TNF-α, produced primarily by activated neutrophils and macrophages, induces the release of other pro-inflammatory cytokines, including IL-6 and IL-1β, and accelerates tumorigenesis [27–29]. Moreover, NF-κB contributes to the resolution of inflammation, which in turn disrupts NF-κB function [25]. Similarly, the adaptive immune response allows recognition of non-self-antigens and the synthesis of antibodies because of cell or body fluid. Cell-mediated immunity consists of T lymphocytes that recognize antigens treated by antigen-presenting cells through their T cell receptors (TCR) [30, 31]. These cells participate in innate immune responses that require NF-κB activation. Thus, T cells are activated by TCR to proliferate and differentiate effector factors required for functional and humoral NF-κB-mediated immunity consisting of B lymphocytes that directly detect B antigens through B cell receptors [31, 32]. With the development of immunotherapy, inhibiting tumor progression by using immunological principles is complicated, and it involves many factors, among which the TIME is an important factor affecting the therapeutic effect. In our study, after THC administration, the expression level of inflammatory-related factor TNF-α, IL-2, and IL-10 increased in the peripheral blood of 4T1 tumor-bearing mice with subcutaneous and lung metastasis, whereas the expression level of IL-1β decreased. Immunohistochemistry and WB experiments showed that the cells in tumor and spleen tissues of tumor-bearing mice could be observed. THC can downregulate the expression level of CYP1A1, NF-κB, and PD-L1 proteins in tumor tissues. FCM analysis of tumor tissues and spleen cells in tumor-bearing mice showed that THC can increase the proportion of CD8 + T cells in spleen and tumor tissues, reduce the abundance of MDSCs, and promote the transformation of M1-type macrophages. These results indicate that THC can regulate the TIME, improve immunosuppression, and play an anti-breast cancer role in vivo through the CYP1A1/NF-κB/PD-L1 axis.

Our study demonstrated that THC plays an anti-breast cancer role by regulating the immune microenvironment of breast cancer cells through the CYP1A1/NF-κB axis in vivo and in vitro and inhibiting the proliferation and metastasis of breast cancer cells.

## Data Availability

The datasets used/ and or analysed during the current study are available from the corresponding author on reasonable request.
